# Allogeneic transplantation of programmable cells of monocytic origin (PCMO) improves angiogenesis and tissue recovery in critical limb ischemia (CLI): a translational approach

**DOI:** 10.1186/s13287-018-0871-8

**Published:** 2018-04-27

**Authors:** Rouven Berndt, Lars Hummitzsch, Katharina Heß, Martin Albrecht, Karina Zitta, Rene Rusch, Beke Sarras, Andreas Bayer, Jochen Cremer, Fred Faendrich, Justus Groß

**Affiliations:** 1 Department of Cardiaovascular Surgery, University Hospital of Schleswig-Holstein, Campus Kiel, Arnold-Heller-Str. 3, Hs 18, D-24105 Kiel, Germany; 2 Department of Anesthesiology and Intensive Care Medicine, University Hospital of Schleswig-Holstein, Kiel, Germany; 3 Institute of Neuropathology, University Hospital Münster, Münster, Germany; 4 Department of Applied Cell Therapy, University Hospital of Schleswig-Holstein, Kiel, Germany

**Keywords:** Programmable cells of monocytic origin, Monocytes, Stem cells, Peripheral arterial disease, Critical limb ischemia, Cell therapy, Regenerative medicine

## Abstract

**Backround:**

Employing growth factor-induced partial reprogramming in vitro, peripheral human blood monocytes can acquire a state of plasticity along with expression of various markers of pluripotency. These so-called programmable cells of monocytic origin (PCMO) hold great promise in regenerative therapies. The aim of this translational study was to explore and exploit the functional properties of PCMO for allogeneic cell transplantation therapy in critical limb ischemia (CLI).

**Methods:**

Using our previously described differentiation protocol, murine and human monocytes were differentiated into PCMO. We examined paracrine secretion of pro-angiogenic and tissue recovery-associated proteins under hypoxia and induction of angiogenesis by PCMO in vitro. Allogeneic cell transplantation of PCMO was performed in a hind limb ischemia mouse model in comparison to cell transplantation of native monocytes and a placebo group. Moreover, we analyzed retrospectively four healing attempts with PCMO in patients with peripheral artery disease (PAD; Rutherford classification, stage 5 and 6). Statistical analysis was performed by using one-way ANOVA, Tukey’s test or the Student’s *t* test, *p* < 0.05.

**Results:**

Cell culture experiments revealed good resilience of PCMO under hypoxia, enhanced paracrine release of pro-angiogenic and tissue recovery-associated proteins and induction of angiogenesis in vitro by PCMO. Animal experiments demonstrated significantly enhanced SO_2_ saturation, blood flow, neoangiogenesis and tissue recovery after treatment with PCMO compared to treatment with native monocytes and placebo. Finally, first therapeutic application of PCMO in humans demonstrated increased vascular collaterals and improved wound healing in patients with chronic CLI without exaggerated immune response, malignant processes or extended infection after 12 months. In all patients minor and/or major amputations of the lower extremity could be avoided.

**Conclusions:**

In summary, PCMO improve angiogenesis and tissue recovery in chronic ischemic muscle and first clinical results promise to provide an effective and safe treatment of CLI.

**Electronic supplementary material:**

The online version of this article (10.1186/s13287-018-0871-8) contains supplementary material, which is available to authorized users.

## Background

Critical limb ischemia (CLI) results from insufficient supply of blood due to arterial stenosis/ occlusion, vessel trauma or vasoconstriction because of catecholamine therapy and shock. Incidence of CLI is ~ 220 new cases per 1 million per year and 30% of these patients will not have options in open or endovascular revascularization [1–5]. For these patients, major limb amputation remains as the only lifesaving treatment option. In response to the significant need for new strategies to prevent tissue damage after CLI and consecutive major limb amputation, there have been numerous studies investigating different strategies in cell-based therapy for inducing neoangiogenesis in ischemic tissues. These previous studies mostly investigate the application of bone marrow-derived hematopoietic stem cells in ischemic tissue but also include a wide range of embryonic stem cells, mesenchymal cells, skeletal myoblasts, and endothelial progenitor cells [6–9].

Among them, dedifferentiated progenitor cells of myeloid precursors have been described as a promising strategy for cell transplantation, inducing regeneration of chronic ischemic tissue [10, 11]. However, most approaches in cell therapy have yet failed to generate a significant impact on clinical practice because of several unsolved issues [12, 13]. First, transplanted cells are generally insufficiently assumed in ischemic tissue and longtime resilience is usually not featured and investigated in angiogenic therapy. Second, usually a high number of cells for cell transplantation and thus a feasible source in clinical practice is necessary. Third, in most cases transplanted cells may not have been directly involved in forming vascular structures but possibly contribute to neoangiogenesis and vascular remodeling by paracrine mechanisms, which have not been investigated in detail so far [12, 14].

Considering these aspects, programmable cells of monocytic origin (PCMO) provide several characteristics rendering them potentially valuable for solving the major limitations of cell transplantation therapy in CLI. As recently reported by our group, peripheral blood monocytes can be partially dedifferentiated into CD14+ progenitor cells with multipotent properties resuming cell division and downregulated factors known to promote their terminal differentiation while retaining other characteristics of the myelomonocytic lineage [10, 15]. PCMO have been reported to provide strong expression of CD14, CD90 and CD115 and weak expression of CD123 whereas other monocyte and stem cell markers such as CD16, CD34, CD117 and CD135 were low or undetectable [11, 15]. Dedifferentiated monocytes have been shown to be responsive to inductive stimuli that directed differentiation into somatic cells of all three germ layers [15]. Successful tissue engineering from PCMO as well as significant improvement of ischemic heart muscle in animal studies after treatment with PCMO has supported cautious optimism that multipotent dedifferentiated monocytes could also be used for angiogenic therapy [11, 15].

Based on our previous research [11, 15, 16], we hypothesized that PCMO can contribute to neoangiogenesis and recovery of ischemic muscle in a paracrine manner. Here, we describe a translational approach and provide evidence from (1) in vitro experiments, (2) animal studies and (3) first healing attempts in men, that PCMO exert therapeutic pro-angiogenic effects and might be suitable for the treatment of CLI.

## Methods

### Isolation and generation of PCMO from mouse and human origin

Mononuclear cells were isolated from peripheral blood of male C57BL/6 donor mice by (Charles Rivers Laboratories, Wilmington MA, USA) density gradient centrifugation. Cells (1.3 × 10^7^/cm^2^) could adhere to the bottom of tissue culture flasks for 1 to 2 h in RPMI medium containing 10% fetal calf serum (FCS), 2 mM glutamine, 100 U/mL penicillin and 100 mg/mL streptomycin (all from Invitrogen, Karlsruhe, Germany). Nonadherent cells were removed by gentle washing with phosphate-buffered saline (PBS) and were cultured for 4 days in ‘dedifferentiation medium’ consisting of RPMI 1640-based medium with 140 μM ß-mercaptoethanol, 20 ng/ml murine monocyte/macrophage colony-stimulating factor (M-CSF) (R&D Systems, Wiesbaden, Germany) and 0.4 ng/ml murine interleukin (IL)-3 (R&D Systems). On day 4, the cells (from now termed PCMO) were washed with PBS and harvested mechanically. Characterization of cell lineage was performed by flow cytometry. Antibodies were directly conjugated with either phycoerythrin (CD3, CD34, CD45, CD80, CD86, CD90, and CD117, all from Beckmann Coulter, Krefeld, Germany; CD13, CD123, CD135 from BD, Heidelberg, Germany), FITC (CD19, Beckmann Coulter), or PC5/PE-CyTM5 (CD14, Beckmann Coulter).

Human PCMO were generated by leukapheresis products from five healthy donors (D1–5) and provided by the Department of Transfusion Medicine (University Hospital Schleswig Holstein, Kiel). Mononuclear cells were isolated by density gradient centrifugation (1.3 × 10^7^/cm^2^) and allowed to adhere to the bottom of tissue culture flasks for 1 to 2 h in RPMI medium 1640 containing 10% human AB-Serum (Lonza, Verviers, Belgium), 2 mM glutamine, 100 U/ml penicillin and 100 mg/ml streptomycin (all from Invitrogen, Karlsruhe, Germany). Nonadherent cells were removed by gentle washing with PBS and cultured for 4 days in ‘dedifferentiation medium’ consisting of RPMI-based medium with 140 μM ß-mercaptoethanol, 5 ng/ml human M-CSF (R&D Systems, Wiesbaden, Germany) and 0.4 ng/ml human IL-3 (R&D Systems). On day 4, the cells were washed with PBS and harvested mechanically. Characterization of cell lineage was also performed by flow cytometry as previously reported [10]. PCMO for clinical application were generated in accordance with the European Union, Good Manufacturing Practice (EU GMP) guidelines.

### Induction of hypoxia in PCMO cell cultures

**Fig. 1:**
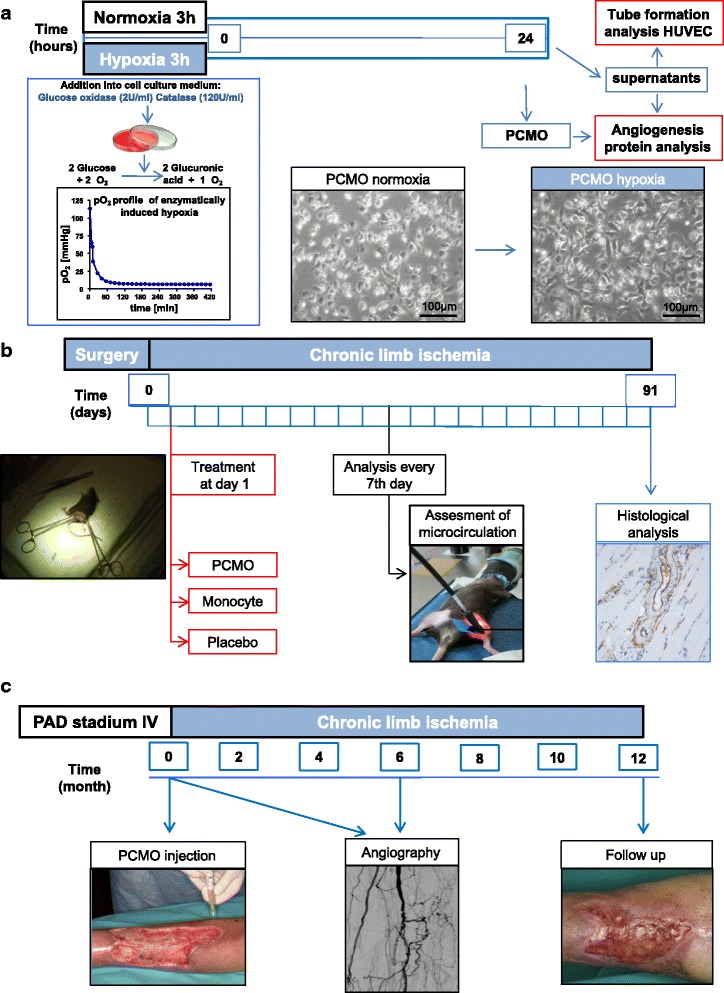
Experimental protocol includes (**a**) cell culture experiments of PCMO evaluating the potential pro-angiogenic effect in vitro, (**b**) animal studies examining the potential effect of treatment with PCMO in contrast to application of native monocytes/placebo group and (**c**) a series of individual healing attemps in patients with peripheral arterial disease (Rutherford, stage 5 and 6) without further curative treatment options. *HUVEC* human umbilical vein endothelial cells, *PCMO* programmable cells of monocytic origin

Simulation of ischemic conditions in vitro was performed by using our recently described enzymatic hypoxia model [17, 18] (Fig. 1a). A series of pretests prior to the major experiments evaluated the optimized duration of transient hypoxia (3 h) and the observation interval (24 h) considering the increase of ischemia-inducible factors in PCMO (Additional file 1: Figure S1) and progression of cell damage during CLI [17, 18].

Employing glucose oxidase (Sigma-Aldrich, Schnelldorf, Germany; final concentration 4 U/ml) and catalase (Sigma-Aldrich, Schnelldorf, Germany; final concentration 240 U/ml) in DMEM high-glucose medium with 1% FCS (PAA, Coelbe, Germany) in combination with a standard six-well system (NUNC, Roskilde, Denmark), partial pressure of oxygen (pO2) in the culture medium and its temporal decline after the addition of glucose oxidase and catalase was measured by using a flexible probe (LICOX® CMP Oxygen Catheter, Integra, Plainsboro, NJ, USA). Concentrations of glucose within the culture media were determined using the Fehling’s method. Fehling’s reagents I and II (Sigma-Aldrich, Schnelldorf, Germany) were mixed with the samples and boiled in a water bath for 15 min. Absorbance was determined at 495 nm using an enzyme-linked immunosorbent assay (ELISA) reader (Tecan, Crailsheim, Germany) with Magellan software v1.1. Standard curves were created from known concentrations of glucose.

### Isolation of RNA and polymerase chain reaction

Cells were washed twice with phosphate-buffered saline (Sigma-Aldrich, Schnelldorf, Germany) and suspended in RLT buffer. Isolation of RNA was done with the Qiagen RNeasy minikit according to the manufacturer’s protocol (Qiagen, Hilden, Germany). RNA concentrations in the samples were quantified with a spectrophotometer at 260 nm. Purity of RNA was assessed by the 260/280 nm ratio. A total of 200 ng total RNA was used to produce cDNA by a reverse transcription kit (Applied Biosystems, Carlsbad, CA, USA) using random hexamer primers. A 2 μl sample was used as a template for PCR experiments in a final volume of 20 μl. All PCR experiments were performed with DNA Taq Polymerase from Solis BioDyne (Tartu, Estonia). Primers were chosen based on the available literature about ischemia-induced gene expression in monocytes/macrophages [19] (Additional file 1: Figure S1). The primer sequences are given in an additional Table (Additional file 2: Table S1). Negative controls were performed by omitting the respective input cDNA. PCR products were separated on 2.5% agarose gels followed by ethidium bromide staining and were visualized by ultraviolet transillumination. For evaluation of gene expression levels, gels were scanned and the respective bands were densitometrically analyzed with the software ImageJ (v1.41o; National Institutes of Health, Bethesda, MD, USA). Values are depicted as relative densitometric units.

### LDH cytotoxicity assay

The colorimetric Cytotoxicity Detection KitPLUS (Roche, Mannheim, Germany) was used for the quantification of cell damage by measuring lactate dehydrogenase (LDH) activity released from cultured cells (Additional file 3: Figure S2). Preparation of samples and measurements were performed according to the specifications of the manufacturer. Briefly, cell culture supernatants were collected 24 h after hypoxia. For the evaluation of total LDH activity, cell lysis was performed with 2% Triton X-100 (Roth, Karlsruhe, Germany). The 100-μL samples were measured per well of a 96-well plate at 492 nm using an ELISA reader (Tecan, Crailsheim, Austria) with Magellan software v1.1 (Tecan, Crailsheim, Austria), and values of absorbance were depicted as arbitrary units (a.u.).

### Proteome profiling arrays

Proteome profiling arrays (R&D Systems, Minneapolis, MN, USA) were performed according to the protocol of the manufacturer. After culturing and treating PCMO as described above (*2.1*), equal amounts (40 μg) of cell lysates (for intracellular proteins) and 150 μl of cell culture medium (for secreted proteins) from each donor (*n* = 5) were pooled and applied to the respective array membrane (Fig. 1a). Expression levels of 55 angiogenesis and tissue recovery associated proteins were evaluated by densitometric analyses of the arrays using the ImageJ 1.41 software (ImageJ, NIH, Bethesda, MD, USA).

### Isolation of human umbilical vein endothelial cells (HUVEC)

Human umbilical vein endothelial cells (HUVEC) were freshly isolated from umbilical cords as described previously [20] and maintained in a humidified atmosphere (5% carbon dioxide/95% air) at 37 °C in endothelial cell growth medium (ECGM) (Promocell, Heidelberg, Germany) supplemented with 4 μl/ml of endothelial cell growth supplement, 0.1 ng/ml epidermal growth factor, 1 ng/ml basic fibroblast growth factor, 90 μg/ml heparin, 1 μg/ml hydrocortisone (all from Promocell) and 10% heat-inactivated fetal bovine serum (Thermo Fisher, Schwerte, Germany), and maintained as primary cell culture. For angiogenesis, cells were detached with a mild cell detachment solution (Accutase, San Diego, CA, USA) and seeded as indicated for the experiments.

### Endothelial tube formation with PCMO-conditioned cell culture supernatants

HUVEC were resuspended in the respective PCMO-conditioned cultured media (2 × 10^5^cells/ml), and 50 μl were seeded into angiogenesis imaging chambers (Ibidi GmbH, Muenchen, Germany) containing Matrigel (Fig. 1a). After 2 h, photomicrographs of the cells were taken every hour until 7 h. The analyses compared PCMO supernatants hypoxia-conditioned vs. PCMO supernatants non-hypoxic-conditioned from five donors (D1–5) and pooled samples from all donors. Angiogenesis parameters (number of segments, nodes and meshes) from each picture were evaluated with the angiogenesis analyzer of the ImageJ software (v1.410) at six different time points (2 h–7 h), as previously described [21, 22].

### Animal studies

To evaluate whether PCMO induce neoangiogenesis in vivo, a hind limb ischemia mouse model was introduced for the evaluation of putative paracrine effects of the PCMO prior to the first clinical application (Fig. 1b). Seven-week-old male C57BL/6 mice (Charles Rivers Laboratories, Wilmington MA, USA) weighing 26 ± 4 g were housed individually with fresh food and water ad libitum during the holding time. To avoid transport stress, the animals had been observed for 10 days before the series of experiments began. All mice underwent surgery and consecutive hind limb ligature and were distributed among three experimental groups: the first group (*n* = 24) was treated with PCMO (1 × 10^6^ cells), the second group (n = 24) was treated with native monocytes (1 × 10^6^ cells) and the third group (n = 24) was the placebo group without treatment.

Hind limb ischemia was induced in mice using a slight modification of a previously described method [23] to simulate long-term ischemia and to avoid complete necrosis of the hind limb. All animal experiments were performed under ketamine/xylasine anesthesia (100/16 mg/kg body weight). Microsurgical-trained physicians performed all surgical procedures. After the hind limbs were shaved and cleaned (with 70% ethanol and betadine), a longitudinal skin incision of ~ 1 cm was made in the groin region, the connective tissue was removed and the femoral artery, vein and nerve were exposed. Two 7/0 polypropylene ligatures were applied on the femoral artery between the distal branch of the artery epigastric superficialis and the trifurcation of the femoral artery into the descending genicular, popliteal, and saphenous branches. Mice were then allowed to recover under a heating lamp to prevent hypothermia. For analgesia, all animals received subcutaneous injections of buprenorphine hydrochloride (Temgesic®, 0.1 mg/kg body weight) prior to surgery and on the first postoperative day. One day after surgery, allogeneic cell transplantation (1 × 10^6^ cells/ 2 ml aqua ad) was performed by intramuscular (i.m.) injection at the anteromedial and posterior muscle compartment of the left thigh. Animals were visually examined for toe necrosis and/or toenail loss by a blinded observer at intervals of 7 days and for a total of 91 days.

### Microlight guide spectrophotometry (O2C)

In vivo characterization of microcirculation in mice was performed by micro-lightguide spectrophotometry (O2C; LEA-Medizintechnik GMBH, Gießen, Germany), as previously described [24]. Oxygen saturation (SO_2_) and capillary blood flow was noninvasively assessed by O2C at intervals of 7 days and for a total of 91 days.

Animals were narcotized with ketamine/xylasine as described in *2.1* and fixed on the back with outstretched legs. Measuring points were marked to ensure reproducibility of the measurements. Probes were positioned between the thighs extensor, adductors and lower leg extensor (Fig. 1b). Pretests (not shown) were performed to evaluate and avoid inter-observer variability. The Doppler data were normalized as the mean ratio of ischemic to contralateral limb measurements.

### Histological and immunofluorescence analysis

Paraffin-embedded tissue slides with thickness of 5 μm from the hind limbs of the mice were stained with hematoxylin-eosin (HE) and myoglobin (1:1000, DakoCytomation, Glostrup, Denmark) to evaluate the morphometrics of the ischemic muscle (Fig. 1b).

Immunohistochemistry was carried out using the avidin-biotin-peroxidase technique. Antibodies against CD34 (1:200, Abcam, Cambridge, United Kingdom) and CD105 (1:4000, Abcam, Cambridge, United Kingdom) were applied to identify emerging endothelial vascular tubes, capillaries and arterioles, as previously described [25, 26]. The secondary antibody was a mixture of anti-rabbit and anti-goat mouse sera. Diaminobenzidine (Leica Biosystems, Nussloch, Germany) served as chromogen. Vascular density (VD) as well as tissue degeneration (decrease of muscle fibers and increase of steatosis) were determined by scanning ten randomly selected high power fields (HPF) at ×400 magnification and in a blinded manner. Positive capillaries (CD105 staining) and arterioles (CD34 staining) were quantified separately and multiplied to the number of positive cells in the same HPF. This resulted in two distinct scores, presented as mean ± SD. Steatosis was expressed as percentage of adipocytes versus the total number of cells per HPF. Muscle degeneration was expressed as number of muscle fibers per mm^2^.

### Retrospective analysis of four clinical individual treatments with PCMO in men

The Institutional Ethics Committee approved the first therapy with PCMO in humans pursuant to clinical individualized treatments in patients with chronic peripheral artery disease (PAD; Rutherford classification, stage 5 and 6), ankle brachial index < 0.6, no option of surgically revascularization and no further treatment option, except major amputation (independently determined by two senior surgeons). All clinical individualized treatments were in accordance with the 1964 Helsinki declaration and its later amendments or comparable ethical standards. Individual patient consent for the individualized treatment was obtained prior to therapy. PCMO for clinical applications were generated in the Clinic of Applied Cell Therapy (University Hospital Schleswig Holstein, Kiel, Germany) in accordance with the EU GMP guidelines. PCMO (8 × 10^8^ cells/ 10 ml aqua ad) were injected 3.5 cm deep at 30 sites into the anteromedial and posterior muscle compartment of the lower leg (Fig. 1c). For detailed clinical presentation and demographic data, please refer to additional Table S2 (Additional file 4: Table S2). All patients were supervised in our outpatient unit. Aftercare check-up followed at 4-week intervals and control angiography was performed after 6 months (Fig. 1c). To evaluate the effectiveness and safety of clinical application, a retrospective analysis of patient data and outcome was performed in the present study.

### Statistical analyses

All values were expressed as mean ± standard deviation (SD). Categorical variables are presented as frequency distributions (n) and simple percentages (%). Data were analyzed with GraphPad Prism version 7.0 for Windows (GraphPad Software®; San Diego, CA, USA). The sample size for the experimental design was calculated using the free G*power 3.1-software (http://www.gpower.hhu.de). Statistical analysis of the results was performed by one-way analysis of variance () for repeated measures and Tukey’s test or Student’s *t* test, when appropriate. Equality of group variances was analyzed by the Brown-Forsythe test. A *p* value less than 0.05 was considered significant.

## Results

### PCMO release pro-angiogenic and tissue recovery-associated proteins under hypoxia

**Fig. 2:**
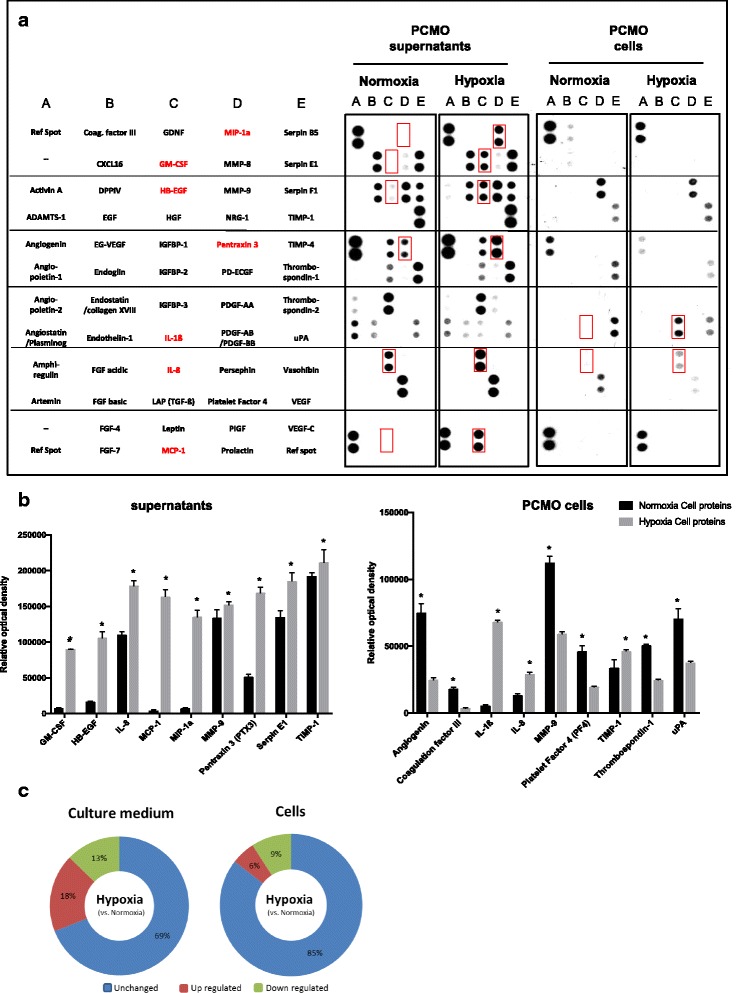
Proteome profiling arrays (**a**) representing 55 proteins involved in angiogenesis and tissue regeneration were performed with the respective PCMO samples. *Red rectangles* represent upregulated proteins in supernatants of PCMO cell culture or cells by more than 25%. The nine most regulated proteins (**b**) in supernatants under hypoxia were: granulocyte-macrophage colony-stimulating factor (GM-CSF), heparin-binding EGF-like growth factor (HB-EGF), interleukin-8 (IL-8), monocyte chemotactic protein 1 (MCP-1), macrophage inflammatory protein 1-alpha (MIP-1α), matrix metalloproteinase (MMP-9), pentraxin-related protein (PTX-3), serpin E1 and metallopeptidase inhibitor 1 (TIMP-1). The most regulated proteins (b) within PCMO under hypoxia were: angiogenin, coagulation factor III, interleukin-1 beta (IL-1β), interleukin-8 (IL-8), matrix metalloproteinase (MMP-9), platelet factor 4 (PF4), metallopeptidase inhibitor 1 (TIMP-1), thrombospondin-1 and urokinase-type plasminogen activator (uPA). The cell culture experiments showed a hypoxia-induced upregulation (**c**) by more than 25% of 10/55 (18%) proteins in supernatants and 3/55 (6%) proteins within the PCMO (*p < 0.05). *PCMO* programmable cells of monocytic origin

To evaluate whether transient ischemic conditions could influence the expression pattern of PCMO for proteins involved in angiogenesis and tissue recovery, cell lysates (for intracellular proteins) as well as cell culture media (for secreted proteins) of PCMO were collected 24 h after hypoxia and normoxia, respectively. Human proteome profiling arrays (Fig. 2a) representing 55 proteins involved in angiogenesis and tissue regeneration were performed with the respective samples and showed a hypoxia-induced upregulation by more than 25% of 10/55 (18%) proteins in culture medium and 3/55 (6%) proteins within the PCMO cells. All investigated proteins and their expression levels (relative optical density) are summarized in additional Table S3 (Additional file 5: Table S3).

Various proteins were upregulated in cell culture supernatants as well as in the cell itself by more than 25% under hypoxic conditions. In detail, the most upregulated proteins in culture supernatants were: monocyte chemotactic protein 1 (MCP-1; 43-fold, *p* < 0.0001), macrophage inflammatory protein 1-alpha (MIP-1α; 15-fold; *p* < 0.0001), granulocyte-macrophage colony-stimulating factor (GM-CSF; 11-fold; *p* < 0.0001), heparin-binding EGF-like growth factor (HB-EGF; 6-fold; *p* = 0.0003) and pentraxin-related protein (PTX-3; 3-fold; *p* = 0.0028) while within PCMO, upregulation of the interleukins IL-1β (IL-1β; 12-fold; *p* < 0.0001) and IL-8 (IL-8; 1.25-fold; *p* = 0.0002) dominated (Fig. 2b + c).

### Supernatants from PCMO induce in vitro angiogenesis and show donor-specific potency

**Fig. 3:**
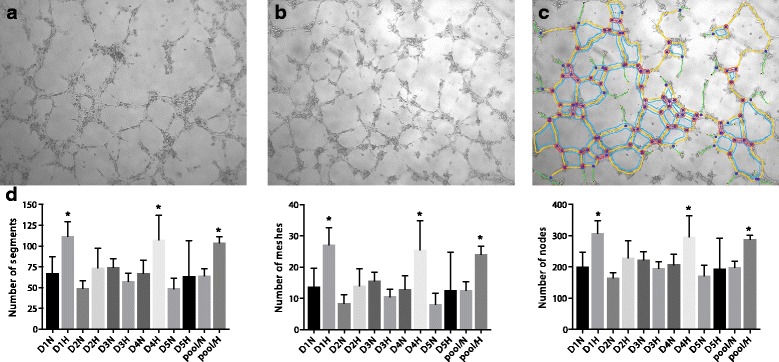
Representative phase contrast pictures of tubular network on Matrigel formed by (**a**) PCMO hypoxic-conditioned and (**b**) PCMO non-hypoxic-conditioned cell culture supernatants derived from donor D1, D2, D3, D4, D5 and pooled donor samples. **c** Corresponding extracted skeletons of tubular networks identifying segments (*yellow color*), mesh (*blue color*) and nodes (*red color*). Branches are marked *green* (not analyzed). Quantitative assessment (**d**) was performed by the angiogenesis analyzer of the ImageJ software. (*H* hypoxic conditioned, *N* non-hypoxic conditioned; *p < 0.05 compared to D1N, D2N, D2H, D3N, D3H, D4N, D5N, D5H, pool/N)

Tube formation assays for angiogenesis were performed employing HUVEC that were cultivated with PCMO cell culture supernatants (hypoxic vs. non-hypoxic conditioned) on Matrigel-coated dishes. Endothelial tube formation showed that supernatants of PCMO, both hypoxic and non-hypoxic conditioned, enabled the formation of vessel tubes in vitro (Fig. 3). Parameters of angiogenesis (number of segments, nodes and meshes) were evaluated and demonstrated a significant enhancement of tube formation by hypoxic-conditioned supernatants of donor D1 and D4 compared to PCMO supernatants cultured from donor D2, D3 and D5 (Fig. 3). Likewise, hypoxic-conditioned pooled samples of all five donors generated significantly more nodes, segments and meshes in comparison to PCMO supernatants from donor D2, D3 and D5 (Fig. 3). For detailed results of the one-way ANOVA, please refer to Table S4–6 (Additional files 6, 7, 8: Table S4–S6).

### PCMO improve microcirculation in a hind limb ischemia mouse model

**Fig. 4:**
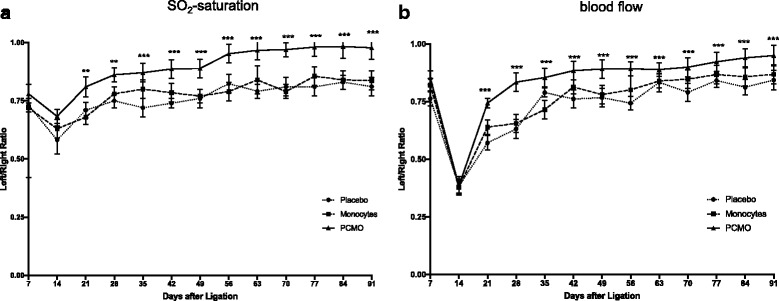
Temporal evolvement (91 days) of (**a**) SO_2_ saturation and (**b**) blood flow in the hind limb ischemia mouse model after injection of PCMO, native monocytes and placebo treatment. Changes of SO_2_ saturation and blood flow were expressed as the ratio of the ischemic (*left*) and non-ischemic (*right*) hind limb (left/right ratio). (**p < 0.003; ***p < 0.0001). *PCMO* programmable cells of monocytic origin

After inducing hind limb ischemia in mice, microcirculation was assessed by micro-lightguide spectrophotometry at 7-day intervals. Changes of the tissue oxygen saturation (SO_2_) and blood flow were expressed as the ratio of the ischemic (left) and non-ischemic (right) hind limb (left/right ratio). From day 21, a significantly higher SO_2_ ratio was observed in the PCMO group in contrast to the monocytes (PCMO: 81.7% ± 4.2% vs. monocytes: 68.4% ± 3.1%; *p* < 0.0001) and the placebo group (PCMO: 81.7% ± 4.2% vs. placebo: 71.4% ± 3.4%; *p* < 0.0001) (Fig. 4a). Blood flow decreased at day 14 and increased again at day 21 in all three experimental groups (Fig. 4b). At day 21 a significant improvment of blood flow was obserevd in the PCMO group in contrast to the monocytes (PCMO: 74.3% ± 2.1% vs. monocytes: 63.3% ± 3.1%; *p* < 0.0001) and the placebo group (PCMO: 74.3% ± 2.1% vs. placebo: 57.5% ± 3.2%; *p* < 0.0001) (Fig. 4b). At day 91, a significantly higher SO_2_ ratio (PCMO: 97.8% ± 4.9% vs. monocytes: 83.7% ± 4.1%; *p* < 0.0001) and blood flow restoration (PCMO: 94.6% ± 4.4% vs. monocytes: 86.4% ± 4.2%; *p* < 0.0001) were detected in the PCMO group in contrast to the monocytes and the placebo group (placebo SO_2_ ratio: 81.1% ± 3.9%; placebo blood flow: 84.3% ± 4.2%; PCMO vs. placebo: *p* < 0.0001). No significant differences in SO_2_ ratio (*p* = 0.163) and blood flow recovery (*p* = 0.058) were observed between the native monocytes and the placebo group during the observation period (Fig. 4).

Further, in all three groups, toe necrosis and/or toenail loss were observed in the ischemic leg. However, the prevalence of necrosis was significantly lower in the PCMO group compared to the monocytes (PCMO: 0.227 ± 0.53 vs. monocytes: 1.045 ± 1.29; *p* = 0.045) and the placebo group (PCMO: 0.227 ± 0.53 vs. placebo: 1.364 ± 1.32; *p* = 0.003).

### PCMO prevent tissue degeneration in ischemic muscle in vivo

**Fig. 5:**
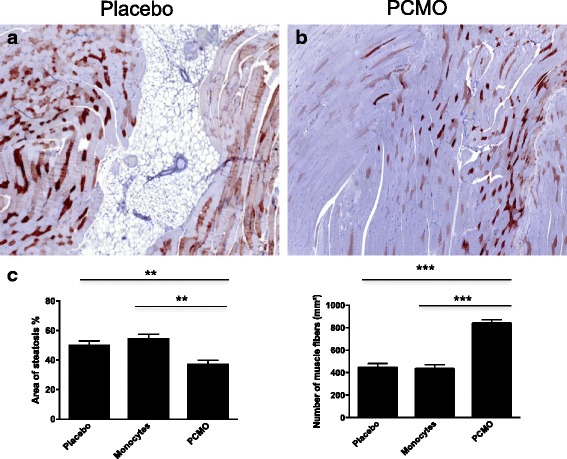
Representative histological examination (myoglobin and HE staining) of muscle atrophy in the hind limb ischemia mouse model (average value ± standard deviation). **a** After 91 days, significantly less signs of steatosis and (**b**) a significantly higher volume of muscle fibers were observed in the PCMO group compared to (**c**) the monocytes and placebo group pointing toward a putative regenerative effect of PCMO in ischemic muscle (**p ≤ 0.002; ***p < 0.0001). Scale bar 100 μm. *PCMO* programmable cells of monocytic origin

Chronic hind limb ischemia in mice was verified by myoglobin staining, quantification of steatosis and the number of muscle fibers as described before [27, 28] (Fig. 5). All three animal groups presented strong accumulation of myoglobin and adipocytes as indication for the chronic muscle degeneration process (Fig. 5a). Nevertheless, the PCMO group showed significantly less muscle atrophy (as seen in Fig. 5b) indicated by the significantly lower decrease of muscle fibers (PCMO: 839.2 ± 135 vs. monocytes: 434.2 ± 163.6; *p* < 0.0001) and steatosis (PCMO: 37.6% ± 9.97% vs. monocytes: 54.65% ± 12.97%; *p* = 0.001) in contrast to the monocytes and placebo group (placebo muscle fibers: 444.6 ± 159.8; PCMO vs. placebo: *p* < 0.0001; placebo steatosis: 50.5% ± 11.06%; PCMO vs. placebo: *p* = 0.002). Comparision of the monocytes and placebo group showed no significant difference between the number of muscle fibers (*p* = 0.961) and accumulation of adipocytes (*p* = 0.362).

### Treatment with PCMO enhance neoangiogenesis in ischemic muscle in vivo

**Fig. 6:**
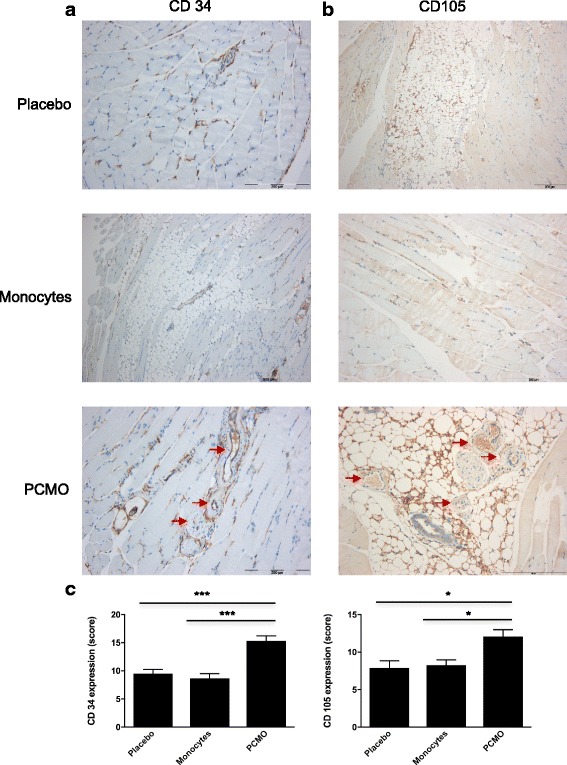
Representative staining of (**a**) arterioles with endothelial antibodies against CD34 and staining of (**b**) capillaries with endothelial antibodies against CD105 (*red arrows*). **c** Quantification of CD34+ arterioles and CD105+ capillaries demonstrated significantly increased vascular density in the PCMO group at 91 days compared to the native monocytes and placebo group, indicating PCMO have some pro-angiogenic properties (average score value ± standard deviation; *p ≤ 0.038; ***p < 0.0001). Scale bar 200 and 500 μm. *PCMO* programmable cells of monocytic origin

After the animal experiments, immunohistochemical staining with endothelial antibodies against CD34 and CD105 was performed to evaluate the effect of PCMO on neoangiogenesis in the hind limb ischemia mouse model (Fig. 6). Immunohistochemical examination revealed a significantly enhanced number of CD34+ arterioles (score PCMO: 15.31 ± 4.04 vs. score monocytes: 8.79 ± 3.78; *p* < 0.0001) and emerging CD105+ capillaries (score PCMO: 12.05 ± 4.21 vs. score monocytes: 8.25 ± 3.22; *p* = 0.030) in the ischemic muscle after transplantation of PCMO in contrast to the monocytes and the placebo group (score placebo CD34+ arterioles: 9.63 ± 3.40; PCMO vs. placebo: *p* < 0.0001; score placebo CD105+ capillaries: 7.91 ± 4.22; PCMO vs. placebo: *p* = 0.038).

### Treatment with PCMO prevents limb amputation, induces vascular growth and improves wound healing in men

**Fig. 7:**
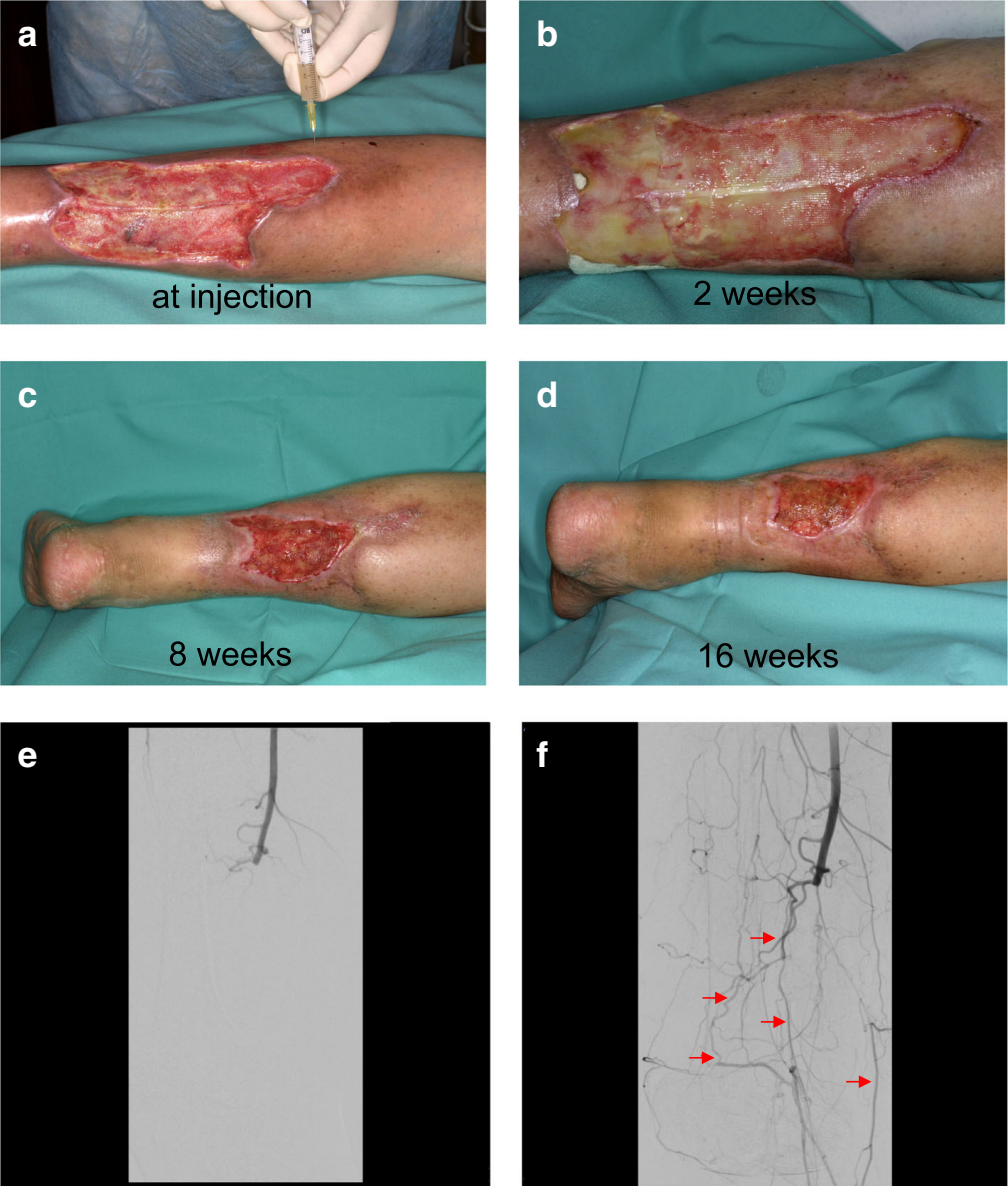
**a**-**d** Documentation of wound healing after treatment with PCMO for the first 16 weeks. After 12 months, improved wound healing was described in all four patients (wound area prior vs. post PCMO application: 21.67 cm^2^ ± 3.58 vs. 13.27 cm^2^ ± 3.53 cm^2^). **e**-**f** After 6 months, the angiography revealed increased growth of vascular collaterals (*red arrows*) in all patients after treatment with PCMO (number of vascular collaterals prior vs. post PCMO application: 8.75 ± 1.71 vs. 13.75 ± 3.30)

Four patients with chronic PAD and no further curative treatment options except major amputation were treated with injections of PCMO into the anteromedial and posterior muscle compartment of the lower leg (Fig. 7). No minor and/or major amputations of the lower extremity were performed in all four patients. After 6 and 12 months no exaggerated immune response, malignant processes or extended infection were reported. The retrospective analysis of data is briefly summarized in Table 1 and additional Table S2 (Additional file 4: Table S2). After 6 months, the angiography revealed increased growth of vascular collaterals in all patients after treatment with PCMO (vascular collaterals prior PCMO treatment: 8.75 ± 1.71 vs. post PCMO treatment: 13.75 ± 3.30) (Fig. 7). Data of the aftercare check-ups (at 12 months) also demonstrated improved wound healing (wound area prior PCMO treatment: 21.67 cm^2^ ± 3.58 vs. post PCMO treatment: 13.27 cm^2^ ± 3.53 cm^2^) and 6-minute walk test results in all patients, as presented in Table 1.

**Table 1:** Patient characteristics after 12 months and vascular collaterals detected in the control angiography after 6 months

Patient	Age (y), gender	6-minute walk test (m) pre-treatment	6-minute walk test (m) post-treatment	Decrease of leg ulcer (%)	Ankle brachial index post-treatment	Increase of vascular collaterals (%)	Immunological events
1	57 m	97 m	323 m	42	0.7	71	None
2	68 m	150 m	324 m	46	0.8	70	None
3	84 f	107 m	157 m	73	0.6	45	None
4	71 f	110 m	211 m	64	0.7	66	None

Area of leg ulcers and numbers of vascular collaterals were expressed as percentage decrease or increase pre- and post-treatment with PCMO

## Discussion

During the last decade, different stem cell entities have been investigated in experimental and small clinical trials as a putative therapy for chronic PAD and ischemic tissue regeneration [6–9]. As previously described by our group, blood monocytes undergo a dedifferentiation process when stimulated with a combination of GM-CSF and IL-3 and become PCMO characterized by a unique surface phenotype consisting simultaneously of lineage and stem cell markers [10, 11]. Although the mechanism by which PCMO achieve their permissive state is still uncertain, it has been shown that the combination of GM-CSF and IL-3 is critical in this process and PCMO apparently share several markers of stem cell renewal and maintenance [10, 15]. Our group has also provided evidence at mRNA and protein level that critical pluripotency regulators such as octamer-binding transcription factor 4 (OCT4), homeobox protein NANOG and MYC are reactivated in PCMO and induce cell plasticity [15]. It has been assumed that PCMO could potentially influence angiogenesis and tissue recovery in a paracrine manner [11]. Here, we show that PCMO improve angiogenesis and regeneration in ischemic muscle in mice and humans and that their properties have the potential to solve several issues in the field of cell therapy.

The present cell culture experiments revealed high resilience of PCMO under hypoxia implying suitability for cell transplantion into chronic ischemic tissues. PCMO showed a paracrine release of pro-angiogenic factors and hypoxic-induced upregulation of proteins pointing toward a putative ischemia-mediated secretion of angiogenetic proteins. Both, hypoxic and non-hypoxic conditioned supernatants of PCMO enabled vascular tube-forming in vitro indicating a significant enhancement of vascular tubes by hypoxic-conditioned supernatants. The hind limb ischemia mouse model demonstrated significantly increased neoangiogenesis, functional and morphometric recovery of chronic ischemic muscle after transplantation of PCMO. Healing attempts in human tend to confirm these results without exaggerated immune response, malignant disease or extended infection after 12 months of follow-up.

One major limitation in allogeneic cell therapy is that cell products for transplantation can only be derived from adult stem cell populations such as bone marrow-derived stem cells or from human embryonic stem cells generated by somatic cell nuclear transfer or from cells with induced pluripotency [15]. Accordingly, biological, economic, and ethical restrictions of all potential cell sources impede the clinical feasibility. Whereas, whole blood as a feasible source for the generation of PCMO can be retrieved easily by a minimally invasive procedure or acquired from waste products in blood donation [10, 15]. Further, our recently described protocol enables short culture times of PCMO with enhanced cell plasticity (4 days) [15]. Thus, PCMO appear as a clinical feasible and ethical responsible source for cell therapy as well as tissue engineering.

Moreover, it is also a well-known problem in cell therapy that transplanted cells are poorly retained in ischemic tissues and subsequently lose their functionality [29]. Interestingly, ischemia-inducible factors increased relatively late in PCMO (Additional file 1: Figure S1) and thus our experiments demonstrated a certain resilience of PCMO without substantial cell damage under hypoxia as demonstrated by marginally raised levels of LDH at 2 h, 3 h and 4 h (Additional file 3: Figure S2). The morphology of PCMO was slightly altered and cells represented a more spherical and attached phenotype (as seen in Additional file 3: Figure S2) but no significant increase in activity of LDH was measured under hypoxia.

Despite of the well-known fact that monocytes can partially differentiate into endothelial-like cells [30, 31], it has been hypothesized that neoangiogenesis induced by cell transplantation may have been influenced by paracrine secretion of pro-angiogenic effectors rather than a direct involvement in vascular structures [32, 33]. The five most upregulated proteins under hypoxia were GM-CSF, HB-EGF, MIP-1α, PTX-3 and MCP-1, and all of them have been strongly associated with angiogenesis and tissue recovery [34–37].

GM-CSF is a cytokine with a wide range of biological effects and its benefit for the recovery of tissue damage has already been described [38]. Accordingly, Zhao and colleagues demonstrated that GM-CSF accelerates wound healing by promoting vascular endothelial growth factor-A (VEGF-A) expression and consecutive the proliferation of endothelial cells [36]. The mediation of the inflammatory response by GM-CSF is also thought to play a decisive role in skeletal muscle regeneration following injury [39]. After muscle injury, neutrophils, monocytes, and macrophages migrate into the damaged tissue, and these cells are believed to tightly regulate the proliferation of myoblasts derived from muscle stem cells (satellite cells) that regulate muscle regeneration [39]. These myoblasts transiently express G-CSFR (granulocyte colony-stimulating factor receptor) following injury and proliferate in response to GM-CSF produced in part by macrophages [39, 40]. Moreover, GM-CSF has many effects on differentiation of myeloid progenitors into heterogeneous populations of monocytes and macrophages [39]. Pearson and colleagues hypothesized that it could have downstream impacts on inflammatory monocytes and macrophages that have not been fully understood yet [41]. It has also been shown that GM-CSF can play a beneficial role by switching inflammatory monocytes to reparative type II macrophages (M2) [42]. In a mouse model of T cell-induced colitis, GM-CSF increased the production of IL-4, IL-10, and IL-13 and decreased the production of interferon gamma (IFN-γ) in lamina propria mononuclear cells. Using a modified Boyden chamber assay, D’abritz and colleagues found that GM-CSF also increased the ability of mononuclear cells to adhere, migrate, and respond to microbial stimuli [42]. Accordingly, intravenous administration of GM-CSF-treated monocytes at the onset of T cell-induced colitis significantly ameliorated the development of disease [39, 42]. Thus, PCMO could have contributed to the muscle regeneration in the present animal experiments and healing attempts by direct stimulation of myoblasts via GM-CSF and the enhancement of M2 macrophages. Finally, it has also been demonstrated that GM-CSF increases vascular collateral flow and conductance, as shown in in a short-term administration of the cytokine in occlusive peripheral artery disease. However, the underlying mechanisms have remained unclear so far [43].

Likewise, MIP-1α and MCP-1 have been identified in the pathways of angiogenesis and in response to vascular inflammation by promotion of accumulation of cells with angiogenic potential [44, 45]. Although MIP-1α has frequently been associated with angiogenesis, its exact (patho)physiological function is still unclear [46]. As reported before, MIP-1α activates chemokine receptor type 5 (CCR5) and c-Jun N-terminal kinase (JNK), extracellular-signal regulated kinase (ERK), or p38 pathway and consecutive leads to down-regulating of miR-374b expression, which promotes VEGF-A expression and subsequently induces human endothelial progenitor cells (EPCs) migration and tube formation [37].

Further, in sites of ischemic results, local production of MCP-1 is enhanced and monocytes are recruited via a chemokine receptor ligand 2 (CCL2) (CCL2 = MCP-1)/CCR2-dependent mechanism associated with a second wave of mononuclear cell recruitment [47]. It may be hypothesized that the recruited monocytes are quickly converted to anti-inflammatory macrophages (M2) at the site of injury where they participate in repair [47, 48]. Studies of mice genetically deficient in MCP-1 or chemokine receptor type 2 (CCR2 = MCP-1 receptor) suggested that although not required for the early mobilization of monocytes, the secondary wave of monocytes recruitment and subsequent stimulation of angiogenesis are dependent upon CCR2 signaling [49].

Moreover, HB-EGF has frequently been reported as a central regulator of angiogenesis and tissue repair [34]. Soluble, mature HB-EGF binds to and activates EGF-receptor, which is a critical molecular key factor to several normal physiological processes including wound healing, reproduction and angiogenesis [43]. In the vascular system, expression of HB-EGF contributes to the remodeling of vascular tissues, and decreases reactive oxygen species (ROS) in human whole blood [49, 50]. Importantly, HB-EGF has been shown to decrease ROS production in stimulated leukocytes, one of the main sources of ROS during tissue injury [51]. Further, it has been demonstrated that HB-EGF restores intracellular adenosine triphosphate (ATP) levels and preserves cytoskeletal integrity in intestinal epithelial cells exposed to hypoxia [45].

The role of PTX-3 in angiogenesis and tissue recovery has been controversial until today [52, 53]. The prototypic long pentraxin PTX-3 orchestrates the recruitment of leukocytes, stabilizes the provisional matrix in order to facilitate leukocyte and stem progenitor cells trafficking, promotes swift and safe clearance of dying cells and of autoantigens and protects the vasculature [52]. Accordingly, Salio and colleagues reported that lack of PTX-3 reduced the number of capillaries in reperfused cardiac tissue, as well as induced a worse outcome in a study of cardiac ischemia [54]. Likewise, PTX-3 has also been described to be a factor that promotes neurogenesis and angiogenesis in neuronal tissue [55]. Despite its beneficial effects in angiogenesis and tissue recovery it could also be demonstrated that high levels of soluble PTX-3 inhibits fibroblast growth factor (FGF) 2-mediated angiogenesis. Endothelium, when exposed to a high density of circulating angiogenic cells (CACs), releases PTX-3 which markedly impairs the vascular regenerative response in an autocrine manner [56]. Therefore, the dose-dependent effect of transplanted cells and subsequent release of angiocrine PTX-3 should be critically assessed for the further development of cell therapies in ischemic disease [55–57].

Collectively, the data of the hypoxia related in vitro experiments indicates that enhanced secretion of GM-CSF, HB-EGF, MIP-1α, PTX-3 and MCP-1 by PCMO could have contributed to the improvement of SO_2_ saturation, blood flow, muscle recovery and neoangiogenesis in vivo. Remarkably, hypoxic-conditioned supernatants of donor D1, D5, as well as pooled supernatants from all five donors showed the strongest increase of angiogenesis in the HUVEC tube formation assays. These results indicate that supernatants of donor D1 and D5 have also influenced the strong pro-angiogenic effect seen in the pooled experiments and that some donors might be better suited to achieve high pro-angiogenic mononuclear cell products than others. Possibly, interindividual immunological effects between the donors or parameters not being represented by the HUVEC tube formation model may have influenced these results. Conceivably the recruitment of CACs and/or circulating mononuclear cells by PCMO could have contributed to the improvement of microcirculation in vivo, as previously described [57]. Industrialized manufacturing and the broad application of somatic cell products, require further research on this important aspect.

In accordance with the in vitro experiments, the animal studies demonstrated a significant improvement of SO_2_ ratio after treatment with PCMO in contrast to native monocytes and the placebo group. Initial decrease of blood flow and velocity could be associated with the first occurrence of tissue damage under ischemia, whereas the significant increase of blood flow and velocity at day 75 seem to be associated with tissue recovery. Accordingly, the VD, indicated by CD34+ and CD105+ vascular endothelium, was significantly higher in mice treated with PCMO. Neither the control group nor the monocytes group presented significant improvements in neoangiogenesis or blood flow restoration. Although monocytes infiltrate dysfunctional tissues and participate in tissue regeneration [38], the present results showed no significant improvement after transplantation of native monocytes in ischemic hind limbs. In comparison, the upregulation of pro-angiogenic proteins in PCMO implies a high angiogenic potency of this cell entity [11, 29]. Remarkably, non-modulated monocytes obviously failed to contribute effectively to neoangiogenesis and tissue recovery indicating the importance of prior reprogramming and preserving a prior developmental stage of monocytes [11, 15]. Our present findings indicate that paracrine secretion of pro-angiogenic factors by PCMO may have contributed relevantly to the improved neoangiogenesis observed in mice and men. Interestingly, the volume of muscle fibers was significantly higher in the PCMO group and significantly less steatosis was observed after the animal experiments. These findings suggest a certain effect of tissue recovery within the PCMO group and correspond with enhanced secretion of GM-CSF and HB-EGF and its influence on extracellular matrix and tissue regeneration [36–38, 40, 50, 51].

Despite its descriptive approach, the small series of PCMO treatment in humans tend to confirm the results from the in vitro and animal experiments. In all patients, minor and major amputations of the lower extremity could be prevented. Angiography after 6 months also demonstrated an increased number of vascular collaterals, thus wound healing was improved and the patients reported a better quality of life. Aftercare at 12 months revealed no exaggerated immune response, malignant processes or extended infection. Nonetheless, future randomized clinical trials should ensure a clear differentiation from other effects and influences such as gait training. Dose dependency, ideal type of application and long-term side effects of transplanted PCMO are future challenges in clinical trials, as demonstrated by the limited experience so far and often disillusioned results from clinical experiences with cell therapy in the past [13, 14].

## Conclusions

In conclusion, PCMO are a feasible source for pro-angiogenic and regenerative somatic cell therapy in CLI. In vitro experiments revealed the pro-angiogenic potential of PCMO and identified five significantly upregulated proteins (GM-CSF, HB-EGF, MIP-1α, PTX-3 and MCP-1) under hypoxic conditions. Animal experiments as well as the first healing attempts in humans showed improved angiogenesis, tissue recovery and clinical outcome without adverse events. The proteins being secreted by PCMO under hypoxia could potentially have contributed in different ways to improved outcome after CLI: first, by regulation of the muscle stem cell proliferation and tissue remodeling (GM-CSF, HB-EGF), second, by the recruitment of circulating mononuclear cells (GM-CSF, MCP-1, PTX-3), third, by switching inflammatory monocytes to reparative type II (M2) macrophages (GM-CSF), fourth, by inducing VEGF-A expression (GM-CSF, MIP-1α). Thus, we propose that differentiated pluripotent programmable cells of monocytic origin (PCMO) should be further characterized for their suitability in (autologous or allogeneic) therapies aiming at the reduction of critical limb ischemia and also ischemia/reperfusion injury in different organs.

## Additional files

Additional file 1 **Figure S1.** Showing the evaluation of hypoxia-induced gene expression in PCMO. (PDF 828 kb)

Additional file 2 **Table S1.** Presenting primers relate to hypoxia-induced gene expression in monocytes/macrophages. (DOCX 308 kb)

Additional file 3 **Figure S2.** Quantification of cell damage by measuring LDH after 1 h, 2 h, 3 h and 4 h and representative images of PCMO cell culture under normoxia and hypoxia. (PDF 674 kb)

Additional file 4 **Table S2.** Presenting patient characteristics prior to treatment with PCMO. (DOCX 18 kb)

Additional file 5 **Table S3.** Presenting human angiogenesis and tissue recovery-related proteins analyzed in PCMO cell culture. (DOCX 813 kb)

Additional file 6 **Table S4.** Results of the tube formation assays for angiogenesis. One-way ANOVA providing the number of segments. (XLSX 51 kb)

Additional file 7 **Table S5.** Results of the tube formation assays for angiogenesis. One-way ANOVA providing the number of meshes. (XLSX 61 kb)

Additional file 8 **Table S6.** Results of the tube formation assays for angiogenesis. One-way ANOVA providing the number of nodes. (XLSX 57 kb)

## Abbreviations

ANOVA: Analysis of variance
ATP: Adenosine triphosphate
CACs: Circulating angiogenic cells
CCL2: Chemokine receptor ligand 2
CCR2: Chemokine receptor type 2
CCR5: Chemokine receptor type 5
CLI: Critical limb ischemia
ECGM: Endothelial cell growth medium
ELISA: Enzyme-linked immunosorbent assay
EPC: Endothelial progenitor cell
ERK: Extracellular-signal regulated kinase
EU GMP: European Union, good manufacturing practice
FCS: Fetal calf serum
FGF: Fibroblast growth factor
G-CSFR: Granulocyte colony-stimulating factor receptor
GM-CSF: Granulocyte-macrophage colony-stimulating factor
HB-EGF: Heparin-binding EGF-like growth factor
HE: Hematoxylin-eosin
HPF: High power fields
HUVEC: Human umbilical vein endothelial cells
IFNγ: Interferon gamma
IL: Interleukin
JNK: C-Jun N-terminal kinase
LDH: Lactate dehydrogenase
M2: Prereparative type II macrophage
MCP-1: Monocyte chemotactic protein 1
M-CSF: Monocyte/macrophage colony-stimulating factor
MIP-1α: Macrophage inflammatory protein 1-alpha
p38: p38 signaling transduction pathway
PAD: Peripheral artery disease
PBS: Phosphate-buffered saline
PCMO: Programmable cells of monocytic origin
PTX-3: Pentraxin-related protein 3
ROS: Reactive oxygen species
VD: Vascular density
VEGF-A: Vascular endothelial growth factor-A
