# Supplementary figures and images for: Allogeneic transplantation of programmable cells of monocytic origin (PCMO) improves angiogenesis and tissue recovery in critical limb ischemia (CLI): a translational approach
Source: Stem Cell Res Ther. 2018 Apr 27;9:117. doi: 10.1186/s13287-018-0871-8 (PMC5921555; doi:10.1186/s13287-018-0871-8)

## PCMO +/- Hypoxia (1h, 2h, 3h and 4h)

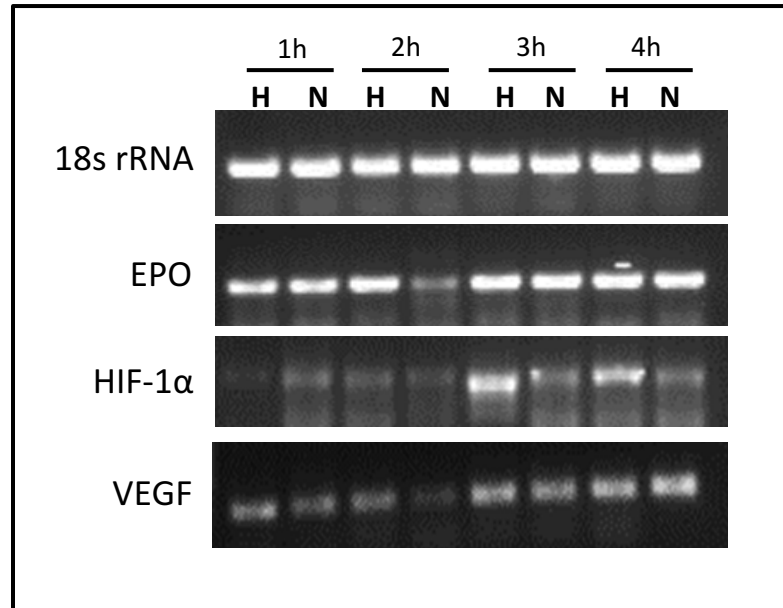

Supplement: Supplementary file 1 — Figure S1. Showing the evaluation of hypoxia-induced gene expression in PCMO. (PDF 828 kb) [file 13287_2018_871_MOESM1_ESM.pdf]

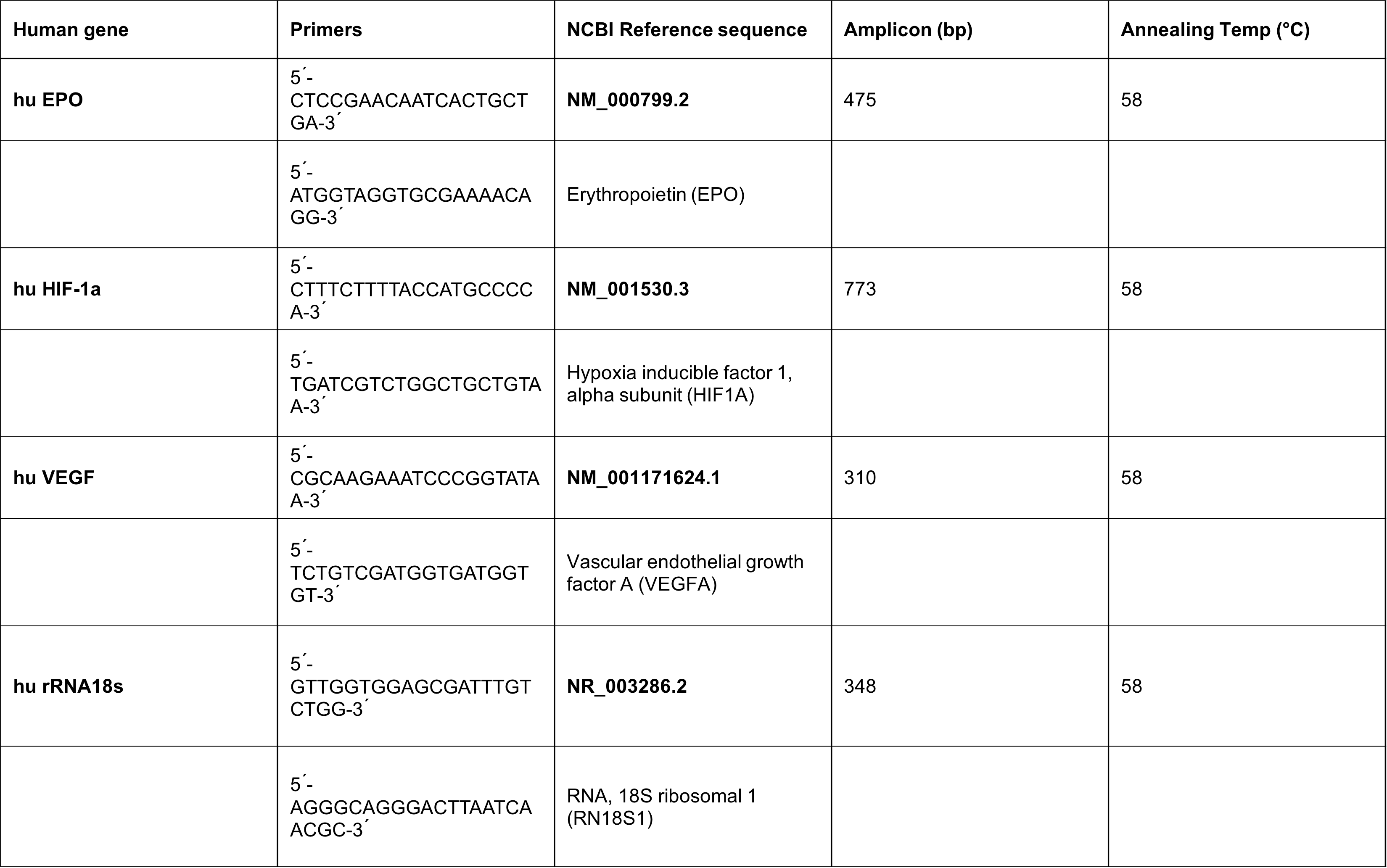


**Table S1:** RNA-Primer sequences

Supplement: Supplementary file 2 — Table S1. Presenting primers relate to hypoxia-induced gene expression in monocytes/macrophages. (DOCX 308 kb) [file 13287_2018_871_MOESM2_ESM.docx]

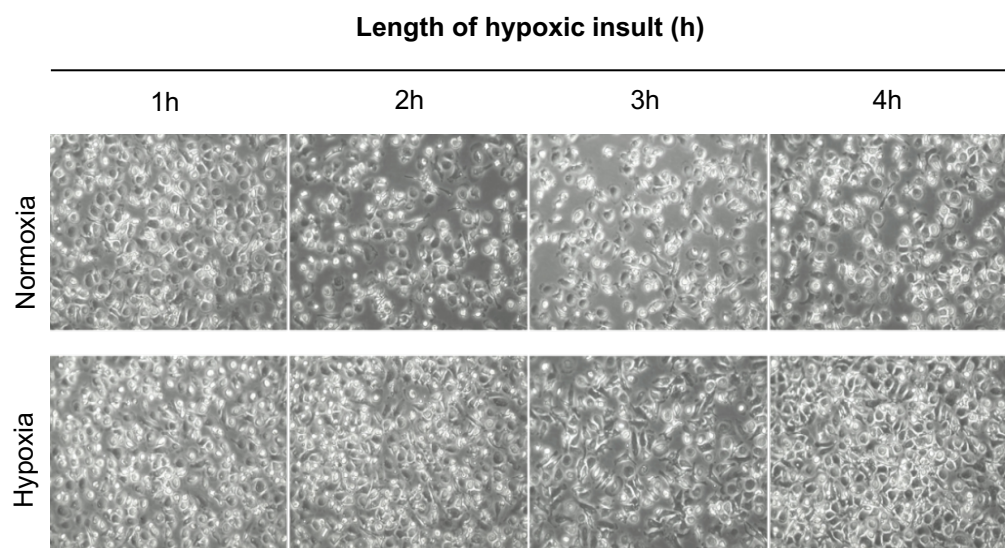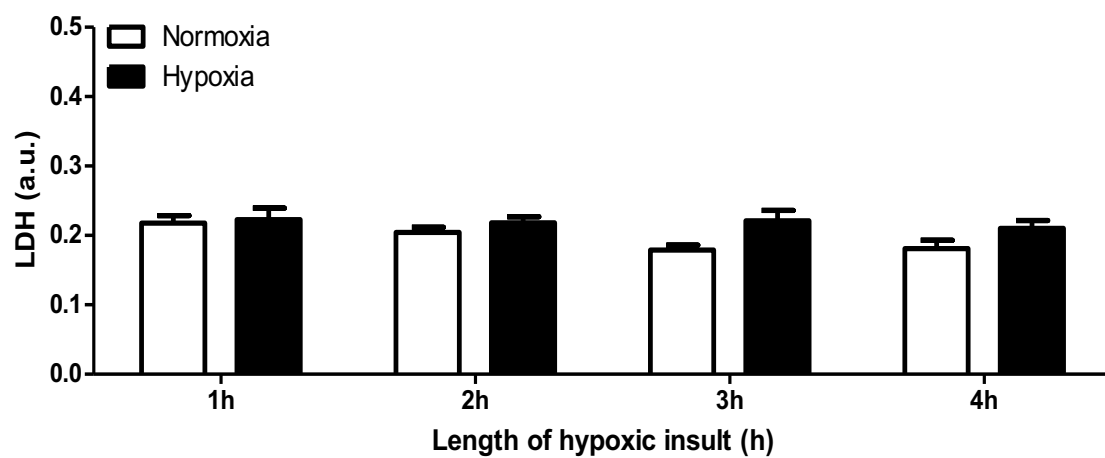

Supplement: Supplementary file 3 — Figure S2. Quantification of cell damage by measuring LDH after 1 h, 2 h, 3 h and 4 h and representative images of PCMO cell culture under normoxia and hypoxia. (PDF 674 kb) [file 13287_2018_871_MOESM3_ESM.pdf]

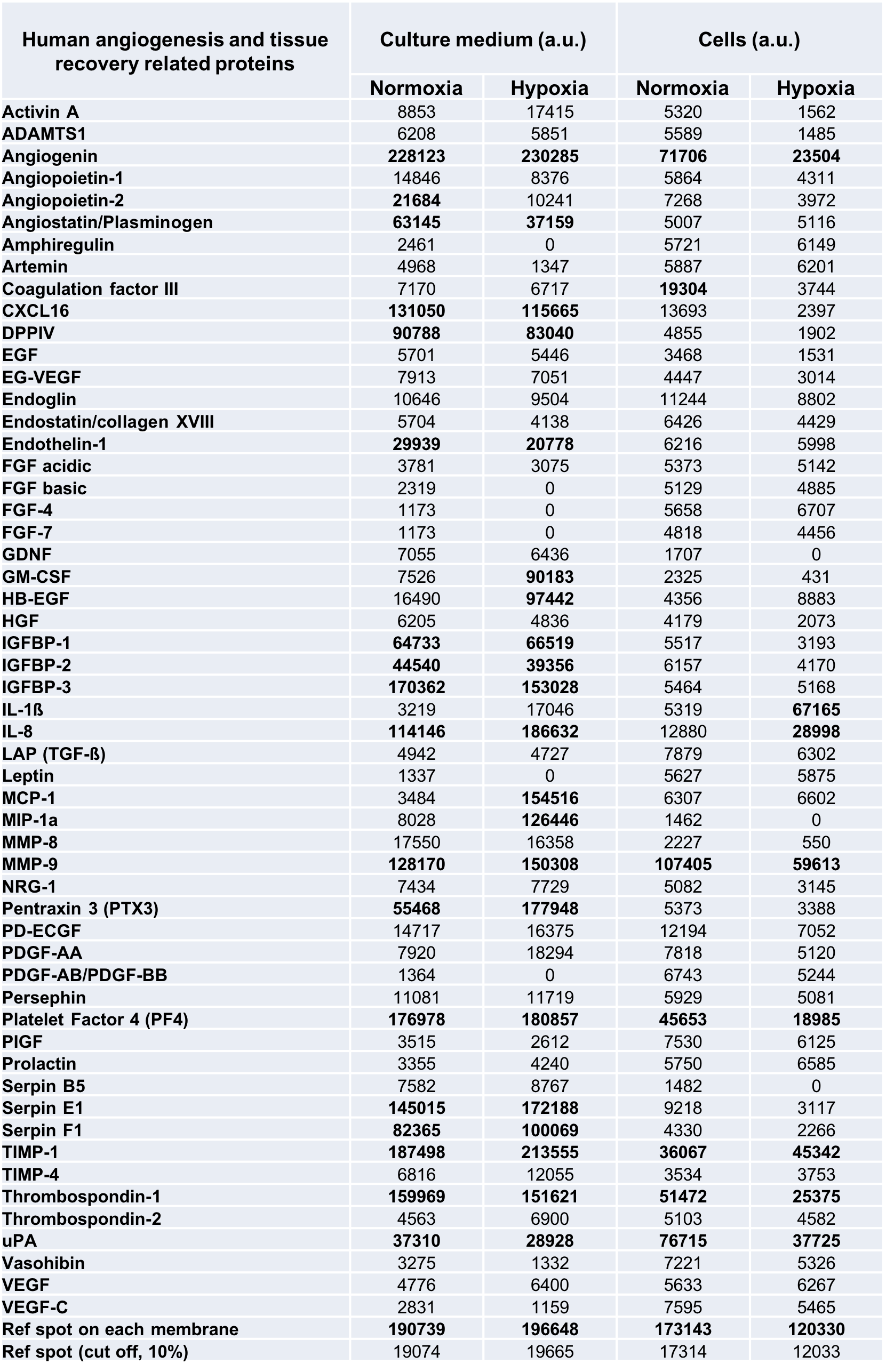

Supplement: Supplementary file 5 — Table S3. Presenting human angiogenesis and tissue recovery-related proteins analyzed in PCMO cell culture. (DOCX 813 kb) [file 13287_2018_871_MOESM5_ESM.docx]
