# Supplementary material for: Allogeneic transplantation of programmable cells of monocytic origin (PCMO) improves angiogenesis and tissue recovery in critical limb ischemia (CLI): a translational approach
Source: Stem Cell Res Ther. 2018 Apr 27;9:117. doi: 10.1186/s13287-018-0871-8 (PMC5921555; doi:10.1186/s13287-018-0871-8)
Supplement: Supplementary file 4 — Table S2. Presenting patient characteristics prior to treatment with PCMO. (DOCX 18 kb) [file 13287_2018_871_MOESM4_ESM.docx]

**Table S2:** *Patient characteristics prior to treatment with PCMO*

| Euroscore 21.23 ± 9.41% | |
| --- | --- |
| Euroscore II 20.84 ± 8.72% | |
| Myokardinfarkt 1 (25%) | |
| CABG 1 (25%) | |
| Peripheral vascular revascularisation 4 (100%) | |
| Renal failure 1 (25%) | |
| Dialysis 1 (25%) | |
| COPD 2 (50%) | |
| Body Mass Index (kg/m^2^) | 25.45 ± 2.36 |
| Low-Density Lipoprotein Cholesterol (mg/dL) | 117.71 ± 26.42 |
| High-Density Lipoprotein Cholesterol (mg/dL) | 63.23 ± 12.31 |
| C-Reactive Protein[^∗^](http://www.sciencedirect.com/science/article/pii/S0002914917300668#tbl1fnlowast) (mg/L) | 21.51 ± 14.24 |
| Systolic Blood Pressure (mmHg) | 142.51 ± 16.24 |
| Diastolic Blood Pressure (mmHg) | 76.34 ± 8.43 |
| Diabetes Mellitus | 1 (25%) |
| **Smoking Status:** | |
| - Never | 0 (0%) |
| - Former | 2 (50%) |
| - Current | 2 (50%) |
| Postmenopausal Status (women) | 2 (100%) |
| Hormone Replacement Therapy (women) | 1 (50%) |
